# Efficacy of ticagrelor in the treatment of stable coronary heart disease: A protocol of systematic review

**DOI:** 10.1097/MD.0000000000022600

**Published:** 2020-11-06

**Authors:** Qing-ning Gao

**Affiliations:** First Ward of Cardiovascular Department, Affiliated Hospital of Jilin Medical University, Jilin, Jilin Province, China.

**Keywords:** efficacy, safety, stable coronary heart disease, ticagrelor

## Abstract

**Background:**

This study will assess the efficacy and safety of ticagrelor in the treatment of patients with stable coronary heart disease (SCHD).

**Methods:**

We will search the following databases for relevant potential studies in Cochrane Library, MEDLINE, EMBASE, Web of Science, Google Scholar, Chinese Biomedical Literature Database, and China National Knowledge Infrastructure. We will search all literature sources from inception to the present without limitations of language and publication status. We will only consider randomized controlled trials on exploring the efficacy and safety of ticagrelor for the treatment of SCHD. Investigators will separately examine studies, collect data and appraise study quality. Data synthesis and analysis will be performed using RevMan 5.3 software.

**Results:**

This study will summarize high quality synthesis of present evidence of ticagrelor for the treatment of SCHD.

**Conclusion:**

The findings of this study will provide evidence to appraise whether ticagrelor is effective for the treatment of patients with SCHD.

**OSF registration number:**

osf.io/fq69u.

## 1 Introduction

Stable coronary heart disease (SCHD) is a leading cause of mortality and morbidity around the worldwide.^[1–3]^ It develops when the major blood vessels that supply blood, oxygen and nutrients to the heart become blockage.^[4,5]^ Thus, such reduced blood flow may lead to chest pain (angina), shortness of breath, and heart attack.^[6,7]^ Several risk factors for such disorder include smoking, high blood pressure, high cholesterol, diabetes, and obesity.^[8–10]^ Patients with SCHD often suffer from very poor quality of life. Thus, it is very important to prevent and treat this condition.

Studies reported that numerous managements are available to treat patients with SCHD, including clarithromycin, adenosine, β-blocker therapy, and ticagrelor.^[11–24]^ Previous studies found that ticagrelor can effectively manage SCHD.^[21–24]^ However, there are not consistent conclusions of ticagrelor for the treatment of SCHD. Therefore, this study will systematically assess the efficacy and safety of ticagrelor for the treatment of SCHD.

## 2 Methods

### 2.1 Protocol registry

This study is reported according to the Preferred Reporting Items for Systematic Review and Meta-Analysis Protocols Statement,^[25]^ and it has been registered on Open Science Framework (OSF) (https://osf.io/fq69u).

### 2.2 Eligibility criteria for study selection

#### 2.2.1 Study types

Only randomized controlled trials (RCTs) investigating the efficacy and safety of ticagrelor for the treatment of SCHD will be included. All other studies, such as case report, case series, review, and non-RCTs will be excluded. In addition, RCTs with sample size of less than 10 will be excluded.

#### 2.2.2 Participant types

Any participants who were diagnosed with SCHD will be included without restrictions of race, sex, and age.

#### 2.2.3 Intervention types

We will include RCTs using ticagrelor as an experimental intervention.

The control intervention includes any treatments, except any forms of SCHD.

#### 2.2.4 Outcome types

Primary outcome is all cause mortality. Secondary outcomes are rate of myocardial infarction, cardiovascular mortality, health-related quality of life, and adverse events.

### 2.3 Search methods

The following databases will be searched from their inceptions to the present: Cochrane Library, MEDLINE, EMBASE, Springer, Web of Science, Google Scholar, Chinese Biomedical Literature Database, and China National Knowledge Infrastructure. All electronic databases will be searched without restrictions of language and publication status. All RCTs on evaluating the efficacy and safety of ticagrelor for the treatment of SCHD will be included. We have presented detailed search strategy for MEDLINE in Table 1. We will adapt similar search strategy to any other electronic databases. In addition, we will also search conference proceedings, clinical registry, and reference lists of included studies.

**Table 1:** Detailed search strategy of MEDLINE.

Number	Search terms
1	coronary heart disease
2	coronary artery disease
3	coronary microvascular disease
4	coronary syndrome X
5	ischemic heart disease
6	nonobstructive coronary artery disease
7	obstructive coronary artery disease
8	stable
9	Or 1–8
10	ticagrelor
11	Brilinta
12	Brilique
13	Possia
14	platelet aggregation inhibitor
15	AZD-6140
16	Or 10–15
17	randomized
18	random
19	randomly
20	blind
21	concealment
22	allocation
23	clinical trial
24	controlled trial
25	study
26	Or 17–25
27	9 and 16 and 26

### 2.4 Data collection

#### 2.4.1 Study selection

Two authors will independently carry out study selection based on the previously designed qualified criteria. We will scan the titles, and abstracts of all searched studies for eligibility for inclusion in this study. After irreverent and duplicated studies are removed, we will review whole manuscripts for further eligibility check. We will note each excluded study with specific reason. We will present the process of study selection in a flow chart. Any disagreements between 2 authors will be solved by a consensus and discussion with the help of a third author.

#### 2.4.2 Data extraction

Two authors will independently perform data extraction using standard data extraction sheet. It includes title, first author, time of publication, location, study design, study setting, sample size, participant characteristics (race, age, sex, so on), randomization, blind, treatment details, comparators, outcome measurements, and funding information. Any disagreements between 2 authors will be solved with the help of a third author through decision.

#### 2.4.3 Missing data management

Any missing or insufficient data will be contacted primary study authors to request that information. If we can not obtain such information, only available data will be analyzed and will be discussed.

### 2.5 Risk of bias evaluation

Two authors will independently assess the risk of bias using Cochrane Risk of Bias Tool. It includes study selection bias, performance bias, detection bias, attribution bias, reporting bias, and other bias. Each item is further judged as high, unclear or low risk of bias.

### 2.6 Data analysis

We will use RevMan 5.3 software for statistical analysis. As for continuous data, the pooled outcome results will be calculated as mean difference or standardized mean difference and 95% confidence intervals (CIs). As for dichotomous data, the pooled outcome results will be presented as risk ratio and 95% CIs. We will carry out *I*^*2*^ test for heterogeneity among eligible studies. *I*^*2*^ ≤ 50% indicates as having low heterogeneity, while *I*^*2*^ > 50% means as having high heterogeneity. If low heterogeneity is identified, we will apply a fixed-effects model. On the other hand, if high heterogeneity is found, we will use a random-effects model. When heterogeneity is low, meta-analysis will be performed if at least 2 studies are included on the same interventions, comparators, and outcome measurements. When heterogeneity is high, we will carry out subgroup analysis to identify any possible reasons that may cause such high heterogeneity. If there is still high heterogeneity after subgroup analysis, we will report outcomes as a narrative summary.

### 2.7 Additional analysis

If sufficient studies are available, we will perform subgroup analysis based on the different study quality, treatments, comparators, and outcome measurements. We will also conduct sensitivity analysis to check the robustness of pooled outcome results by excluding low quality studies.

### 2.8 Reporting bias

When sufficient studies are included, we will also carry out Funnel plot^[26]^ and Egger regression text^[27]^ to explore any possible reporting bias.

### 2.9 Ethics and dissemination

No ethic approval is required in this study, because we will not analyze individual data. The results of this study are expected to be published at a peer-reviewed journal.

## 3 Discussion

SCHD is a very tricky heart disorder, which greatly affects health-related quality of life in such population. Previous studies have reported that ticagrelor has been utilized to treat patients with SCHD effectively. However, its results are still inconsistent. Therefore, this study will systematically explore the efficacy and safety of TBF for the treatment of patients with COPD. The results of this study will provide convinced evidence to the clinical practice and patients, as well as the health-related policy maker.

## Author contributions

**Conceptualization:** Qing-ning Gao.

**Data curation:** Qing-ning Gao.

**Formal analysis:** Qing-ning Gao.

**Investigation:** Qing-ning Gao.

**Methodology:** Qing-ning Gao.

**Project administration:** Qing-ning Gao.

**Resources:** Qing-ning Gao.

**Software:** Qing-ning Gao.

**Supervision:** Qing-ning Gao.

**Validation:** Qing-ning Gao.

**Visualization:** Qing-ning Gao.

**Writing – original draft:** Qing-ning Gao.

**Writing – review & editing:** Qing-ning Gao.
